# Medication Experience and Adherence to Oral Chemotherapy: A Qualitative Study of Patients’ and Health Professionals’ Perspectives

**DOI:** 10.3390/ijerph18084266

**Published:** 2021-04-17

**Authors:** Amparo Talens, Mercedes Guilabert, Blanca Lumbreras, María Teresa Aznar, Elsa López-Pintor

**Affiliations:** 1 Pharmacy Department, Hospital General Universitario de Elda, 03600 Elda, Spain; talens_amp@gva.es; 2 Department of Health Psychology, Miguel Hernandez University, 03202 Elche, Spain; mguilabert@umh.es; 3 Department of Public Health, History of Science and Gynecology, Miguel Hernandez University, 03550 San Juan de Alicante, Spain; blumbreras@umh.es; 4 CIBER de Epidemiología y Salud Pública (CIBERESP), 28029 Madrid, Spain; 5 Pharmacy Department, Hospital Universitario de San Juan de Alicante, 03550 San Juan de Alicante, Spain; aznar_mte@gva.es; 6 Department of Engineering, Area of Pharmacy and Pharmaceutical Technology, Miguel Hernandez University, 03550 San Juan de Alicante, Spain

**Keywords:** medication experience, adherence, oral cancer chemotherapy, qualitative study, antineoplastic agents

## Abstract

Lack of adherence constitutes one of the most important challenges in patients undergoing treatment with oral antineoplastic drugs (ANEO). Understanding cancer patients’ experiences with respect to their medication is key for optimizing adherence and therapeutic results. We aimed to assess the medication experience (ME) in patients with cancer in treatment with ANEO, to describe the barriers and facilitators related to the disease and its treatment and to compare them with the healthcare professionals’ perspectives. We carried out an exploratory qualitative study in the University Hospital of San Juan de Alicante, Spain. Three focus groups and two nominal group discussions were conducted with 23 onco-hematological patients treated with ANEO and 18 health professionals, respectively. The data were analyzed using content analyses and were eventually triangulated. The most impactful aspects in patients’ ME were the presence of adverse effects; lack of information about treatment; beliefs, needs and expectations regarding medications; social and family support; and the relationship with the health professionals. Both patients and professionals agreed on considering the negative side effects and the information about treatment as the main barriers and facilitators of adherence, respectively, although the approaches differed between both profiles. The professionals offered a more technical vision while patients prioritized the emotional burden and motivation associated with the disease and medication. This study allowed us to understand the real-life experiences of patients being treated with ANEO and explore the factors which had an impact on adherence to treatment. This understanding enables professionals to have a positive influence on patients’ behavior and provide individualized care plans. Pharmacists’ assistance is relevant to support patients’ adherence and self-management.

## 1. Introduction

Over the last decade, 25% of antineoplastic drugs were administered orally [1], a per-centage that is increasing. The oral method for the administration of antineoplastic treatment is also the preferred option for both patients and professionals [2,3,4], for its convenience and for enabling a better quality of life [5]. However, lack of adherence constitutes one of the most important challenges in patients treated with oral antineoplastic drugs (ANEO). Previous studies showed heterogeneous results in adherence rate with ANEO, within a range of between 23 and 100% [6], according to the drugs involved and the measurement methods used.

Adherence to general drug therapy treatment is multidimensional and the factors that may influence adherence to chronic oral treatments have been extensively described [7]. Many of these factors are related to the medication experience (ME), in other words, how the drugs influence people’s daily lives [8]. The ME allows us to understand how medication impacts on patients’ beliefs, behaviors, health and wellbeing. Including this experience in clinical decision-making enables patients and professionals to improve their decisions and to optimize adherence and therapeutic results [9].

In patients with cancer, the social and cultural characteristics of the disease can differently impact on the barriers and facilitators of adherence to ANEO. Irwin et al. [10] assessed the factors that conditioned adherence to orally administered treatments in different pathologies and in cancer, finding variables common to all chronic diseases, as well as factors that specifically affected patients being treated with ANEO.

Qualitative study techniques are an ideal tool for understanding ME and to assess its influence in the field of adherence [11]. In addition, these techniques allow the comparison between patients’ beliefs and perspectives and health professionals’ opinions [12,13]. Nevertheless, available studies focused on adherence to ANEO are mainly quantitative [10,14]. In addition, there is lack of evidence regarding the application of qualitative techniques to describe the impact of ME on adherence to ANEO in any type of cancer, since the scarce available studies have evaluated a particular ANEO or a specific type of cancer [15,16].

Understanding and taking into account the experiences of patients with cancer with respect to their medication, as well as the professionals’ points of view, is fundamental for guiding the education and the counselling of these patients and for designing tools to optimize therapy [11].

Therefore, we aimed to assess the ME in patients with cancer in treatment with ANEO, to describe the barriers and facilitators related to the disease and its treatment and to compare them with the healthcare professionals’ perspectives.

## 2. Materials and Methods

### 2.1. Design

We carried out a qualitative study in both cancer patients undergoing ANEO treatment and in healthcare professionals (HCP) involved in their clinical management. The study setting was the University Hospital of San Juan de Alicante (HSJ), southeast Spain, a reference hospital for the majority of medical specialties, which has about 400 beds. We used the focus group [17,18] technique with the patients, because of its inclusive approach which encourages various topics through which the participants can explain their own experiences and respond to what other participants say. In the case of the HCP, we selected a nominal group technique [19], together with an interactive voting system, which allowed us to hierarchically structure the ideas put forward by the participants.

### 2.2. Participants

Patients and HCP were recruited in the Pharmacy Service of the HSJ, using the snow ball [20] recruitment system. Inclusion criteria were patients with onco-hematological cancer undergoing treatment with any oral antineoplastic, who went to collect their medication from the Hospital Pharmacy Service. Patients in a terminal phase (survival prognosis of less than six months) were excluded, as were those that did not know how to/could not express themselves fluently. The inclusion criteria for HCP were having at least 5 years of professional experience in the field of oncology and being active when data collection was undertaken.

### 2.3. Procedure

The study included three sessions with patients and two sessions with HCPs. All the sessions took place during 2019, and were moderated by the author GM, supported by observer personnel. The participants had previously given permission to have their interviews audiotaped.

The patient sessions started with a meeting in which the researchers presented the study and its objectives. The participants then introduced themselves and this was fol-lowed by a group debate. Patients were asked to describe their medication-taking journey from the beginning of the ANEO treatment, highlighting how they had dealt with the medication-taking process in their daily life, and which barriers and enablers they had encountered. A structured script was developed comprising a set of key questions regarding the study topic (Appendix A). This script was based on a prior review of the scientific literature about the factors influencing the lack of adherence of patients undergoing ANEO treatment, as well as the research team’s experience.

In the HCP group, questions were focused more specifically on the two elements that could facilitate or hinder patients’ ANEO medication-taking process (Appendix B). The questions were presented to encourage individual reflection and to compare their personal experiences and points of view regarding each topic.

### 2.4. Data Analysis

The audio recordings from the sessions were manually transcribed, and a content analyses tool was used to synthesize and organize all qualitative information. The ideas put forward were ordered and classified by the researchers into units of analysis, using mutually exclusive categories. Information obtained for each category was classified according to spontaneity (sp), how often the same idea was repeated independently and consistency, whether the same idea was repeated in the different groups. Verbatim descriptions of each unit of analysis were selected based on their relevance, frequency or when there was high agreement between the participants.

Finally, the information (cross-validation) obtained from the contributions of both patients and HCP were triangulated to analyze the degree of consistency of the ideas put forward. In order to make the triangulation if an idea was repeated in one group, we assigned the value of 1; if it was repeated in 2 groups we assigned the value of 2, and so on, up to the total of 5 groups (3 of patients and 2 of healthcare professionals). Likewise, in the case of HCP, according to the prioritization system used, the following values were indicated for each idea: intensity of the recommendation (weighting assigned to each idea by the experts), degree of spontaneity and agreement about the recommendation (coefficient of variation).

### 2.5. Ethical Considerations

Both patients and HCP were verbally informed about the aim and methodology of the study, and they voluntarily accepted taking part in the study without any financial remuneration. Informed consent was received from all the participants and guidelines outlined in the Declaration of Helsinki were followed. This study was approved by the Ethical Committee of Clinical Research of the HSJ, as part of a more complex project, “Predictive model of therapeutic adherence in patients with onco-hematological cancer” Cod. 18/327.

## 3. Results

### 3.1. Characteristics of the Participants, Patients and Professionals

A total of 23 patients and 18 healthcare professionals were finally recruited. Table 1 and Table 2, respectively, show the characteristics of the participants. The age of the patients ranged from 32 to 81 years old and the average age was 62.3 years (standard deviation, SD = 13.3). Of the patients, 69.6% were women. The most represented diagnoses in the patient groups were breast cancer and chronic myeloid leukemia. The average number of years since diagnosis was 3.8 ± 2.5 years. The median time taking oral antineoplastic was 8.5 months with a minimum treatment period of 1.8 months and a maximum of 44 months. In the case of the healthcare professionals, different levels of the hierarchy were represented: head of departments, medical specialists, nursing staff and hospital pharmacy interns, from the following departments; oncology (*n* = 27.8%) pharmacy (*n* = 55.6%) and hematology (*n* = 16.7%).

### 3.2. Patientsߣ Perspectives about Oral Chemotherapy

Based on the opinions of the participants, eight categories emerged: (1) experience with the treatment, (2) polymedication, (3) beliefs about the medication, (4) need for treatment and expectations about effectiveness, (5) information and sources relating to the treatment, (6) medication errors and forgetting to take medication and how to prevent this, (7) adverse effects and consequences of the treatment with ANEO and (8) social, family and professional support.

An executive summary of the main findings for each category is described below, together with a selection of key supporting quotations. Additionally, the results from the three focus groups with patients are shown in Appendix C.

1. Experience with the treatment.

A majority of the patients described an extensive experience (years in treatment) with ANEOs, with an average of approximately six years of treatment and various changes in treatment over the years. The following statement illustrates this:

*“I have been in treatment for eleven years: five and a half years with one medication, about three years with the second and the last two years with another one…”*

2. Polymedication.

This category was understood as the taking of a number of different drugs on a daily basis. In this sense, 100% of the patients interviewed took other drugs together with the ANEOs each day, with some patients taking as many as 15 tablets. This was stated as follows:

*“I take at least nine pills a day, for my diabetes, for my heart…”*

3. Beliefs about the disease and medication.

This category refers to patients’ ideas and thoughts on medication effectiveness and coping methods regarding the disease. Most patients (84%) valued positively the ability to take medication orally rather than intravenously. Despite this, during the sessions, participants adopted different stances about the effectiveness of the medication. Several patients were extremely convinced about the effectiveness of the medication, since, at that point in time, it was giving them good results. Some patients, in contrast, expressed doubts because the disease remained latent, but they preferred to assume this risk over other options. The following statements illustrate this:

*“In my case, which is chronic leukemia, the medication has been fantastic. The oncologist has even reduced the dose…”*

*“(…) You have doubts at the start because the treatment doesn’t cure you, and a transplant will, but seeing the risks of the transplant, well, you don’t even think about it…”.*

Several patients highlighted the importance of maintaining a positive coping style with the disease. In this respect, one participant stated the following:

*“I also think that, if you look at it from a positive perspective, you create an environment where the family don’t get used to the idea of the disease you have either (…). I didn’t want to drag my family down with me…”.*

Finally, the avoidance of the subject of the disease was also a topic mentioned by most participants. One of them said:

*“I think that talking about the disease too much wears you down (…) it sometimes feels good to get it off your chest but I prefer only to do this occasionally”.*

4. Need for treatment.

Most patients agreed that medication adherence was key for overcoming the dis-ease, and highlighted the importance of taking it regularly despite adverse effects and placed it above other concomitant health problems. The need and the belief that the medication is necessary was an argument that overcame not taking it. The following statement illustrates this:

*“You can get things off your chest with your family, the oncologist, your friends but you can never stop taking the medication, ever…”*

5. Information and sources relating to the treatment.

Patients spontaneously agreed that the first and main source of information about treatment was the oncologist or the hematologist, depending on the type of cancer. All participants pointed to them as their reference healthcare professional for questions about the disease and treatment-related problems:

*“It was the oncologist who decided on the treatment and explained to me how to follow it”*

Several patients said that they got more in-depth explanations about their treatment from the hospital’s pharmacy service, and that they used to consult the pharmacist for issues related to potential side effects of the medication:

*“Oncologists make the prescription, pharmacists explain it to us“*

The participants also recognized that on some occasions they used the internet to search for information about their diseases and/or treatments, but they agreed that this was not recommendable:

*“I’ve done it (consult google), but I stopped right away because there’s so much information and you don’t know whether it’s true or not. If anything, it makes you more scared”*

6. Preventing medication errors and forgetting to take medication.

Participants were aware of the importance of taking the ANEO treatment correctly, but they recognized having forgotten to take it or not having taken it exactly as pre-scribed. Two patients stated the following:

*“On occasions, yes, it is true that I have forgotten or I was uncertain about whether I’d taken it or not”.*

*“Maybe I’m not so strict about the time, perhaps I was at the start, but not now, if I go out, I take it later”.*

To prevent forgetfulness, the patients mentioned different methods. The most common was organizing the medication around routines, which the patients called “tricks”:

*“I have to take my medication once every 24 h, and I normally take it at night. If I go out at night and I forget, I’ve normally got medication in my car or in my hand-bag (…)”.*

In addition to these “tricks”, the patients mentioned different electronic devices for remembering, such as mobile phone alarms.

*“My support is the mobile, that’s what reminds me”.*

The support of the family and the people that live with the patients, represented another key stimulus for remembering medication. Finally, the traditional pill box was pointed to by some participants as a fairly effective method to avoid forgetting medication:

*“If you carry a pill box you never forget because, if you’re in any doubt about whether or not you’ve taken a tablet, you look at the pill box and that clears up any doubt…”.*

7. Side effects and consequences of the treatment.

The side effects caused by ANEO drugs was the information category that generated the most discussion among patients, who considered them the main barrier to treatment adherence. The patients described many types of side effects (Appendix C), highlighting most spontaneously those relating to tiredness and a general lack of energy, followed by difficulties in walking and cramps. They complained that nobody had warned them about this kind of negative side effects. The following statement illustrates this:

*“Nobody warned me about this lack of energy, I even went to the emergency department, worried because, in the first cycle, I could barely stand up” (…). When I walk it’s like walking on broken glass”.*

All the participants underlined the considerable impact of the side effects of ANEO medication on their quality of life. Some who were in active employment had to leave their jobs or remain on sick leave, and those that carried on working had difficulties reconciling their medication and their job. There were also several comments about the impact of the adverse effects on the patients’ leisure and free-time activities. These statements reflect this:

*“Now I am retired, it doesn’t bother me, but when I was working, I had to organ-ise the timing of medication because it affected me so much”*

*“I had to give up doing the things I liked the most, such as going for walks or going to the beach. Routines, daily life in general were affected”*

*“Your life changes. In my house, there’s nothing but stairs and, for the first few months, I couldn’t get up them (…). In some ways, this does constrain your normal life, but I’ve tried to carry on as normal”.*

8. Social, family and professional support.

The support of family and friends emerged as one of the main pillars for the success of the treatment. For some patients, social support was not just family but, for example, being able to share experiences about the disease with peers:

*“The most important thing is to share it with your family and friends, it’s comforting”*

*“Yes, I’ve been in an association of patients with the same disease as mine”*

One patient in one of the focus groups, said:

*“These sessions where we can share our experience with other people in the same situation are very helpful”*

In addition, the support of the healthcare professionals and the feeling of being in good hands was key for patients, who also alluded to the easy accessibility of the professionals as another contributing factor.

*“My experience is incredibly good. I had never had health problems and heard everyone complaining, but now, based on my experience, I’ve only got good things to say…”.*

*“They (professionals) phoned me, asking me whether I had any symptoms of appendicitis because an enlarged appendix is supposed to be one of the side effects of my treatment… I’m grateful for that”*

To conclude, some patients mentioned the Hospital Pharmacy Service as a facilitator in accessing treatment, highlighting the care provided by the pharmacists. In contrast, several participants mentioned some negative aspects, such as the long waits and the fact that ANEOs could only be picked up from the hospital, and on a monthly basis.

*“The pharmacy is marvelous, they treat you very well…”*

*“One negative aspect I perceive with the medication is having to come and collect it (...). The pharmacy is only open in the morning and it is an effort for me to come, given that I’ll be on this medication for the rest of my life and I have to come and get it every month… I even have to change my work schedule”.*

### 3.3. Professionalsߣ Perspectives about Oral Chemotherapy

The sessions undertaken with the professionals were focused on the discussion of the barriers and facilitators of adherence to ANEO treatment. The ideas obtained from the nominal groups are shown in Appendix D. A summary of the main findings for each category are described below.

#### 3.3.1. Barriers to Adherence

The most consistent idea among both groups of professionals was the perception of the side effects of ANEOs as the main barrier to treatment adherence. The participants in the sessions pointed out the significant impact of side effects and interactions, in general, without specifying which adverse effects could interfere more with the lack of adherence. Another consistent idea that arose in the discussions consisted of those aspects related to posology and the complexity of therapeutic schedules, and even some characteristics of the medication. For example, most participants highlighted the fact that patients often have swallowing problems due to the size of the tablets. In the case of polymedication, other participants emphasized that the large number of drugs was a barrier *“which can force patient to choose between pills”*.

With respect to the use of the internet as a source of information, the professionals warned about the unreliability and bias in the information offered by many web portals. In this sense, one participant pointed out that: *“Dr. Google is a relevant barrier, patients have to be very careful with it”*.

Finally, participants mentioned other barriers, such as forgetting to take medication or not having symptoms of the disease.

#### 3.3.2. Facilitators to Adherence

Most professionals considered that the main facilitator to adherence to treatment was the adequate provision of information to patients. To achieve this goal, the most consistent ideas among groups were those of giving patients information on the prevention and management of adverse effects, giving them verbal as well as written information and customizing the guidelines to the personal characteristics of each patient. These statements illustrate the key ideas in relation to this:

*“We have to educate patients on the prevention of side effects, giving them oral and written information on how to manage them”.*

*“Information is fundamental for the progressive empowerment of patients so that they can take control of their disease”.*

The awareness regarding the need for medication and the importance of generating a positive belief in the effectiveness of the drug was another facilitator of adherence highlighted by the professionals. This sentence exemplifies this idea:

*“It is important for the patient to see the effectiveness of the drug in order to maintain positive expectations of improvement or at least to be able to remain stable”.*

The professionals also referred to the fact that underlining the importance of the disease contributes to the patients overcoming the obstacles related to the treatment.

The professionals agreed that social and professional support was another relevant facilitator. In this sense, they referred to the importance of the accessibility of healthcare professionals and the generating of a relationship of trust with them. They also highlighted the importance of an active patient follow-up and the role of the support provided by the Hospital Pharmacy Service: *“Patients should be closely followed-up when they collect their medication”.*

Finally, regarding the prevention of forgetting to take medication, the professionals highlighted the establishment of routines as a facilitator, and mentioned the usefulness of various applications, diaries and other electronic devices to avoid this problem.

### 3.4. Cross-Validation

A summary of the spontaneity and consistency between the five groups is shown in Table 3. As we can see, the results of the concordance analysis show that the main facilitators of adherence to ANEOs, highlighted by both patients and professionals (consistency = 5), were information and explanations regarding treatment, and having social, family and professional support. Patients and professionals coincided in considering the adverse effects of the medication as the main barrier to adherence, together with forgetting to take medication (consistency = 5). Both groups also highlighted the importance of different technological devices as facilitators of medication adherence.

## 4. Discussion

The results of this qualitative study provide valuable insights into the ANEO-related attitudes of patients with cancer and their perceptions of adherence barriers and facilitators. The main barriers to adherence identified were the presence of adverse effects; lack of information about treatment; beliefs, needs and expectations regarding medications; social and family support; and the relationship with the health professionals. Some of the categories are known factors associated with non-adherence to ANEOs [10]. In this previous systematic review [10], authors found a positive association between provider relations and adherence, including mainly the doctor and the nursing staff. However, in our study, patients remarked upon the importance of trust in the hospital pharmacist, who can play a relevant role to help patients improve their adherence to oral cancer therapy.

Patients’ opinions were in the same direction as the barriers and facilitators perceived by the professionals, although the approaches between both profiles were different. In general, the professionals offered a more technical vision, focused on providing precise information to achieve compliance with the medication and prevent adverse effects, while patients prioritized the emotional burden and motivation associated with the disease and medication. The professionals included terms such as “posology”, “adverse effects-interactions”, “guidelines” or “schedules”, while patients referred to “support”, “accessibility”, “routines”, “trust” or “practicality”. This different vision between patients and professionals with respect to ANEOs has been reported previously by other authors [12,13], and suggests that the two approaches are required to design efficient interventions that guide patients’ education and counselling and improve adherence.

Presence of adverse effects was the category that generated a great discussion by both patients and professionals. The professionals referred to them as the main barrier to compliance and patients specifically detailed their own experiences and expressed their concern about the impact of the adverse effects on their work and leisure time and how they affected their quality of life.

Patients stated that they were willing to deal with the adverse effects, prioritizing the ANEO treatment over any other treatment. This contrasts with previous evidence that suggests that chronic patients use diverse avoidance strategies to avoid adverse effects, with a consequent impact on adherence, such as unreported voluntary dosage changes, inappropriate use of other drugs or even discontinuing the treatment [6,21,22]. These differences could be related to a greater awareness about the need for medication among patients with cancer and the importance of maintaining positive expectations about the effectiveness of the treatment, as explained by both patients and professionals, which raises the tolerance threshold to the adverse effects with respect to other chronic diseases [23,24,25]. In addition, Irwin et al. [10] indicated that in patients with cancer, having positive expectations about the effectiveness of the medication was a factor associated with adherence.

Lack of information about the treatment was also a category that the majority of patients described as a barrier to adherence. Verbrugghe et al. [15], in a qualitative study carried out in Belgium with 30 patients with different types of cancer from five hospitals, indicated that informing patients about possible adverse effects improved adherence to treatment. Regnier-Denois et al. [12], in a qualitative study about adherence to orally administered capecitabine, evaluated the perceptions of 42 patients, suggesting that clinicians emphasized the need of compliance with treatment rather than providing practical information about the treatment. Encouraging the development and implementation of strategies to give patients an early education about the possible side effects associated with ANEOs and how to manage them according to patients’ experiences will contribute to increased adherence to treatment.

In relation to the sources of information, patients firstly considered healthcare professionals as the most reliable, and although they acknowledged having consulted the internet occasionally, this generated even more uncertainty and concern. A good relationship between patients and healthcare professionals is one of the most frequently reported facilitators in the bibliography [9,26]. The oncologist was the reference person for patients but they stated that, when they were doubtful about the medication, or had any adverse effects, they usually went to the hospital pharmacist. Timmers et al. [27] indicated a multi-disciplinary approach as a tool for improving the care of patients being treated with ANEOs, urging hospital pharmacists to contribute to improve adherence. Previous studies have described a positive impact of the hospital pharmacist on improving symptoms reported by patients with cancer and supporting patients in the management of ANEO treatment [28,29]. However, some patients indicated, as a barrier to accessing the medication, the opening hours of the Hospital Pharmacy Service, which sometimes interfered with their work schedule or other routines, causing delays in collecting the medication. Other authors associated forgetting to pick up the medication as a cause of missing doses [30].

Another barrier to adherence to treatment described by both professionals and patients was polymedication. All the patients reported taking several drugs, in addition to ANEO, and described the number of treatments as a barrier. The professionals indicated the complexity of the schedules and dosage, and the physical characteristics of the drugs, as barriers. Irwin et al. [10] showed contradictory results regarding to the influence of these factors on patients in treatment with ANEOs. Geissler et al. [31], in a multicenter observational study that included 2546 patients with chronic myeloid leukemia from 63 countries, showed greater adherence in patients whose treatment consisted of a single daily dose.

Patients reported to be adherent to ANEO, probably due to peer pressure, as it has previously shown [12,13]. However, they recognized the occasional forgetting of some doses, which constituted a frequent reason for unintentional non-adherence. Muluneh et al. [24], in an observational study carried out with 93 patients in treatment with ANEO, showed that 30% of patients acknowledged occasionally missing a dose of their medication.

The professionals also agreed on indicating forgetting medication as one of the main barriers to adherence, and all groups highlighted the establishment of routines as a facilitating element, although with different approaches among the professionals and the patients. The professionals mentioned diaries, apps and other sophisticated electronic devices, while the patients used simpler methods such as mobile alarms and traditional pill boxes. Interventions based on reminders are very effective to increase adherence to treatment [32]. Patients also relied on family members and friends to remind them about doses. In this regard, the family and friends’ support and the ability to share experiences with other patients were highlighted as an important pillar for patients, who even expressed their gratitude for the experience shared in the focus groups of this study.

Length of treatment was another important barrier considered by the patients, but it was not referred to by the professionals. For some pathologies [33], it has a positive influence on adherence; however, for patients with cancer, the duration of the treatment has been identified as a barrier, as shown in other studies [14,34].

Given that in Spain, antineoplastic treatments are included in the pharmaceutical provision of the National Health System, none of the patients included referred to the cost of the treatment at any time during their discussion. Nevertheless, it is considered one of the main barriers to adherence in other countries, due to the high cost of this medication [10,35,36].

This study has some limitations. Firstly, the patients that agreed to take part in this study were probably more adherent than those who were not included. However, the objective of this study was not to evaluate adherence as such, but to understand patients’ experiences with respect to the medication. On the other hand, this study was conducted at a single center, whereby the results cannot be extrapolated for all patients being treated with ANEOs. Despite this, the information obtained may be very valuable for improving the limited knowledge available about the real-life experiences of patients being treated with ANEOs. Moreover, focus groups could have biased participants’ responses due to peer group pressure. However, the moderator tried to minimize this bias by encouraging participation and the free expression of ideas at each session. Finally, it is worth noting that differences in the disease situation of the participants were not considered. This is an aspect that we could bear in mind in subsequent studies, grouping the patients according to their pathology and the stage of the disease. Nevertheless, ANEOs are currently used both in the initial and more advanced stages of the disease, as well as in pathologies with different prognoses.

## 5. Conclusions

The application of qualitative research techniques allowed us to understand the real-life experiences of patients being treated with ANEO and explore the complex interrelationship of personal, social and structural factors which impact on adherence to treatment. The comparison between both patients’ and health professionals’ points of view showed the different approaches among them. It is essential that professionals understand patients’ experiences with the medication to enable them to have a positive influence on their behavior and provide individualized care plans. In addition, pharmacists’ assistance, especially with patients with polypharmacy and chronic diseases, is relevant to support patients’ adherence to oral cancer therapy.

## Figures and Tables

**Table 1:** Patients taking part in focus groups (*N* = 23).

Variables		% (*N*)
Sex	Man	30.4 (7)
	Woman	69.6 (16)
Average age (years) ± SD Median	62.3 ± 13.3 64 (31–82)	
Diagnosis	Breast cancer	30.4 (7)
	Prostate adenocarcinoma	4.3 (1)
	Lung cancer	4.3 (1)
	Ovarian cancer	8.7 (2)
	Chronic myeloid leukaemia	13 (3)
	Ampullary cancer	4.3 (1)
	Renal cancer	4.3 (1)
	Multiple myeloma	17.4 (4)
	Melanoma	8.7 (2)
	GIST ^1^	4.3 (1)
Oral antineoplastic drug (ANEO)	Palbociclib	8.7 (2)
	Abiraterone	4.3 (1)
	Osimertinib	4.3 (1)
	Niraparib	8.7 (2)
	Dasatinib	8.7 (2)
	Capecitabine	13 (3)
	Imatinib	8.7 (2)
	Everolimus	13 (3)
	Sunitinib	4.3 (1)
	Pomalidomide	4.3 (1)
	Lenalidomide	13 (3)
	Dabrafenib+Trametinib	4.3 (1)
	Bosutinib	4.3 (1)

^1^ GIST = gastrointestinal stromal tumor.

**Table 2:** Professionals taking part in Nominal Group (*N* = 18).

	Categories	% (*N*)
Sex	Man	61.1 (11)
	Woman	38.8 (7)
Post	Head of department	16.7 (3)
	Physician/pharmacist Resident/intern	55.6 (10) 11.1 (2)
	Nurse	16.7 (3)
Department	Oncology	27.8 (5)
	Pharmacy	55.6(10)
	Hematology	(3)

**Table 3:** Cross-validation. Consistency between patient and professional groups.

Analysis Categories and Subcategories	Sp ^1^ Professionals	Consistency Professionals	Sp ^1^ Patients	Consistency Patients	Consistency between Groups
**Experience with the treatment**					
Patients expert in the treatment			19	3	3
Patients starting with the treatment			10	3	3
**Polymedication**					
Taking other drugs together with antineoplastics	1	1	26	3	4
Posology	10	2			2
Problems swallowing due to size of tablets	4	1			1
**Beliefs about the medication**					
Doubts about the effectiveness of the medication	-	-	5	3	3
Medication is very effective	-	-	5	3	3
Oral medication rather than intravenous	1	1	22	3	4
Positive coping style	1	1	5	2	3
Avoiding the disease	1	1	4	1	2
Awareness about the disease	1	1			1
**Need for the treatment and expectations about effectiveness**					
Medication is necessary to be able to overcome the disease	1	1	20	3	4
Discontinuing the medication due to side effects	15	2	5	2	4
Discontinuing the medication due to other treatments	6	2	1	1	3
**Information about the treatment and sources**					
Professional informing for first time about the treatment			14	3	3
Explanations about the treatment	11	2	12	3	5
Reference professional in treatment matters			29	3	3
Information from family members who are healthcare professionals			2	1	1
Contradictory information between specialists and primary care doctor	1	1	1	1	2
Oral and written information about the medication	4	2			2
Personalised patient information	2	2			2
Internet to search for more information about the disease	1	1	9	3	4
**Errors, failures, forgetting the medication and their prevention**					
Organisation of the medication based on routines			20	3	3
Reminders about medication by family members			3	3	3
Stimuli/alarms for remembering	2	2	5	3	5
Pill boxes			7	3	3
Blister packs with the days of the week			1	3	3
Forgetfulness	1	1	15	3	4
**Adverse effects and consequences of the treatment**					
Renal and hepatic system disorders			2	2	2
Respiratory system disorders			3	1	1
Development of cardiometabolic diseases			3	2	
Dermatological conditions			4	2	2
Unstable thermal sensation			1	1	1
Tiredness/fatigue/loss of consciousness/lack of energy			8	2	2
Difficulties in walking/cramps			6	3	3
Oral disorders			3	1	1
Musculoskeletal pain			1	1	1
Vision problems			1	1	1
Vomiting			1	1	1
Constipation			1	1	1
Diarrhoea			1	1	1
No side effect			2	1	1
Work-related consequences			8	3	3
Consequences for free-time activities			4	3	3
Consequences for everyday activities			4	2	2
Consequences for physical health			2	2	2
Consequences for mental health			3	3	3
Changes to diet			3	1	1
**Social/family/professional support**					
Sharing experiences about the disease with peers	1	1	2	2	3
Family support with the disease and the treatment	2	2	8	3	5
Feeling in good hands	4	2	11	3	5
Accessibility of healthcare professionals			2	1	1
Care provided by hospital pharmacy department	2	1	22	3	4
Active patient follow-up	3	2			2

^1^ Sp = spontaneity.

## Data Availability

Patient level data, the full dataset and statistical code are available from the corresponding author. Participant consent for data sharing was not obtained but the presented data are anonymized and risk of identification is low.

## Appendix A. Focus Groups Questions

**Table A1:** Focus group questions: adherence to antineoplastic agents.

**With respect to the number and type of drugs that you take:**
*Do you generally take many drugs?*
*Do you consider the medication you are taking to be necessary?*
*Are you getting on well with the oral treatment?*
*Do you think it would be better in intravenous form instead of tablets?*
**With respect to the the moment you started the medication:**
*Who was the first healthcare professional that informed you about the medication?*
*What things did they explain to you?*
*Did you feel that you needed more information about the medication you were being prescribed?*
*Did you search for, before or after, information about the treatment, the effects, benefits, for example, on the internet, from other people, family members, friends* *?*
**With respect to the tricks that you use with the medication, and the things that you do to follow the health professionals recommendations:**
*Have you ever forgotten to take the medication? What do you do when this happens?*
*One thing is forgetting to take medication, but it is something entirely different for you to have voluntarily decided not to take the medication. Could you describe your reasons to do this?*
**I would now like you to talk about a normal day since you have been undergoing this treatment.**
*Do you organise your medication or is there a family member who takes charge of that?*
*In addition to the medication, do you do anything else to maintain your health, for example, physical exercise, walks, activities in general that make you feel well?*
*Do you belong to any association or network of people in a similar situation to yourselves, for example, a group like the one we are in today?*
**Finally, in general, how is your relationship with the healthcare professionals that care for you?**
*Do you think that they are concerned about you, that they care about you?*
*Who is your reference professional for everything regarding the medication?*
*Do you know who you should go to in the event that your medication does not go very well?*
**Is there anything else you would like to tell us about the medication you are taking?**

## Appendix B.

**Table A2:** Questionaire: Nominal Groups Questions.

**Question 1. Main barriers stopping patients from adhering to orally administered antineoplastics, reasons for stopping taking the medication, reasons for discontinuing the treatment.**
*Do you think that it would be better to administer them intravenously instead of in tablet form?*
*Do you feel that your patients need more information about the ANEO medication, that they usually have unresolved doubts?*
*Searching for external information about the treatment, the effects, benefits, for example, on Internet, from other people, family members, friends.*
*Forgetting to take the medication.*
*Side effects, interaction with other drugs, interaction with food, feeling poorly physically, feeling depressed, not being able to keep doing the things I like to do, feeling very well and deciding to discontinue the medication.*
*Not having a reference professional for everything regarding the medication.*
*Not knowing who they should go to in the event that their medication does not go very well.*
**Question 2. As healthcare professionals, what elements might facilitate a patient adhering to orally administered antineoplastics? What do you do, as healthcare professionals, to encourage a positive attitude towards the medication?:**
*Information and explanations about the medication.*
*Help in organising the rest of the medication.*
*Comprehensive information about the health process, medication, other routines for maintaining their health, exercise, walks, activities in general that make patients feel well.*
*Menacing messages about the disease and refusal to take the corresponding medication.*
*Showing real concern for the health of your patients.*
*Recommending other forums for sharing everyday aspects of the disease, including the medication.*

## Appendix C. Nominal Groups Questions

**Table A3:** Focus group results, patients. Analysis categories and subcategories.

Idea/Comment, Professionals	Intensity of the Recommendation	Variance	Sp	Agreement with the Recommendation
**Group 1**				
**Barriers to adherence**				
Side effects	5.0	0.0	7	0.0
Patient not given sufficient information about the medication	4.1	1.0	2	0.2
Fear of adverse reactions	4.0	0.9	1	0.2
Number of pills of drug itself	3.9	0.7	3	0.2
Not understanding guidelines or needs	3.6	1.1	1	0.3
Complexity of therapeutic schedules	3.4	0.8	1	0.2
“Dr. Google”	2.9	1.6	1	0.5
Not feeling effects of disease	2.0	1.7	1	0.9
**Facilitators of adherence**				
Information about adverse effects of the disease-related treatment	5.0	0.0	5	0.0
Generating trust in healthcare professionals	5.0	0.0	3	0.0
Access to professionals, having a reference person to ask about doubts and concerns	4.5	0.3	2	0.1
Social support as a facilitator of therapeutic adherence	4.5	0.3	1	0.1
Preventing side effects. How to manage them	4.1	1.0	2	0.2
Adapting the treatment to the disease. Reducing the number of pills	3.9	0.7	2	0.2
Written indications	3.9	0.7	1	0.2
Being able to share experiences with peers	3.6	0.6	1	0.2
Empowering the patient	3.4	1.4	1	0.4
Adapting the guidelines to personal characteristics	3.4	1.4	1	0.4
Reviewing “collateral” treatments. Deprescription	3.4	0.8	1	0.2
Number of visits. Dispensing of pills	2.9	1.6	1	0.5
Reminders, apps, diaries and other technological devices	2.5	1.1	1	0.5
**Group 2**				
**Barriers to adherence**				
Large number of drugs, “so patient ends up choosing”	4.3	0.2	2	0.1
Side effects	3.9	1.2	5	0.3
Biased information	3.6	1.4	1	0.4
Posology	3.5	2.1	4	0.6
Problems swallowing due to size of tablets	3.4	1.6	4	0.5
Fear of interactions, adverse effects	2.7	1.3	2	0.5
Interaction with other drugs	2.6	1.6	1	0.6
Forgetting taking medication	2.5	1.2	1	0.5
**Facilitators of adherence**				
Personalised patient information	4.8	0.2	1	0.0
Giving patient information both verbally and in writing	4.7	0.2	2	0,0
Written information about the medication	4.5	0.3	1	0.1
Easy posology	4.5	0.3	3	0.1
Support provided by pharmacy department	4.4	0.9	1	0.2
Being insistent during follow-up. Spending a long time on information	4.4	0.3	2	0.1
Treatment during consultation	4.3	0.9	1	0.2
Importance for the patient (awareness)	4.1	0.1	1	0.0
Pharmaco-therapeutic education	4	0.9	1	0.2
That the patient sees the effectiveness of the drug	3.9	1.2	1	0.3
Convenience of picking up medication from the hospital pharmacy office	3.9	1.4	1	0.4
Family support	3.7	1.6	1	0.4
Apps/alarms/reminders	3.1	1.0	1	0.3
Better tolerance than chemotherapy	2.9	1.4	1	0.5

## Appendix D. Nominal Groups Results

**Table A4:** Nominal group results, professionals. Group work ideas.

Idea/Comment, Professionals	Intensity of the Recommendation	Variance	Sp	Agreement with the Recommendation
**Group 1**				
**Barriers to adherence**				
Side effects	5.0	0.0	7	0.0
Patient not given sufficient information about the medication	4.1	1.0	2	0.2
Fear of adverse reactions	4.0	0.9	1	0.2
Number of pills of drug itself	3.9	0.7	3	0.2
Not understanding guidelines or needs	3.6	1.1	1	0.3
Complexity of therapeutic schedules	3.4	0.8	1	0.2
“Dr. Google”	2.9	1.6	1	0.5
Not feeling effects of disease	2.0	1.7	1	0.9
**Facilitators of adherence**				
Information about adverse effects of the disease-related treatment	5.0	0.0	5	0.0
Generating trust in healthcare professionals	5.0	0.0	3	0.0
Access to professionals, having a reference person to ask about doubts and concerns	4.5	0.3	2	0.1
Social support as a facilitator of therapeutic adherence	4.5	0.3	1	0.1
Preventing side effects. How to manage them	4.1	1.0	2	0.2
Adapting the treatment to the disease. Reducing the number of pills	3.9	0.7	2	0.2
Written indications	3.9	0.7	1	0.2
Being able to share experiences with peers	3.6	0.6	1	0.2
Empowering the patient	3.4	1.4	1	0.4
Adapting the guidelines to personal characteristics	3.4	1.4	1	0.4
Reviewing “collateral” treatments. Deprescription	3.4	0.8	1	0.2
Number of visits. Dispensing of pills	2.9	1.6	1	0.5
Reminders, apps, diaries and other technological devices	2.5	1.1	1	0.5
**Group 2**				
**Barriers to adherence**				
Large number of drugs, “so patient ends up choosing”	4.3	0.2	2	0.1
Side effects	3.9	1.2	5	0.3
Biased information	3.6	1.4	1	0.4
Posology	3.5	2.1	4	0.6
Problems swallowing due to size of tablets	3.4	1.6	4	0.5
Fear of interactions, adverse effects	2.7	1.3	2	0.5
Interaction with other drugs	2.6	1.6	1	0.6
Forgetting taking medication	2.5	1.2	1	0.5
**Facilitators of adherence**				
Personalised patient information	4.8	0.2	1	0.0
Giving patient information both verbally and in writing	4.7	0.2	2	0,0
Written information about the medication	4.5	0.3	1	0.1
Easy posology	4.5	0.3	3	0.1
Support provided by pharmacy department	4.4	0.9	1	0.2
Being insistent during follow-up. Spending a long time on information	4.4	0.3	2	0.1
Treatment during consultation	4.3	0.9	1	0.2
Importance for the patient (awareness)	4.1	0.1	1	0.0
Pharmaco-therapeutic education	4	0.9	1	0.2
That the patient sees the effectiveness of the drug	3.9	1.2	1	0.3
Convenience of picking up medication from the hospital pharmacy office	3.9	1.4	1	0.4
Family support	3.7	1.6	1	0.4
Apps/alarms/reminders	3.1	1.0	1	0.3
Better tolerance than chemotherapy	2.9	1.4	1	0.5
